# Civilian gunshot injuries at the emergency department of a Nigerian teaching hospital: patient characteristics, pattern and outcome 2014-2018

**DOI:** 10.11604/pamj.2024.49.27.45070

**Published:** 2024-10-03

**Authors:** Ambrose Rukewe, Temitope Oluwagbenga Alonge, Abayomi Akande, Akinola Ayoola Fatiregun

**Affiliations:** 1 Anaesthesiology Division, School of Medicine, University of Namibia, Windhoek, Namibia; 2 Department of Anaesthesia, University College Hospital, Ibadan, Nigeria,; 3 Department of Orthopaedics Trauma, University College Hospital, Ibadan, Nigeria,; 4 Department of Community Medicine, Faculty of Public Health, University College Hospital, Ibadan, Nigeria,; 5 World Health Organization, Abeokuta office, Abeokuta, Ogun State, Nigeria

**Keywords:** Civilian gunshot injuries, patient characteristics, pattern, outcome

## Abstract

Gunshot injuries (GSI) are a major global public health problem. Our objective was to determine the patient characteristics, pattern and outcome of civilian gunshot wounds at the University College Hospital, Ibadan, Nigeria, from 2014-2018. The data of 232 patients with a diagnosis of GSI during the study period were collected and analyzed using descriptive and inferential statistics. The victims were mostly males (86.6%) and the night shifts had the highest presentations (56%). Majority of the patients (61.2%) were in the age range of 21-40 years. Armed robbery attacks were the most frequent cause of the wounds (78.9%) affecting mostly the upper and lower limbs. Eighteen (7.8%) patients died from injuries to the head, chest, abdomen and multiple parts of the body. There was a significant association between the anatomical location of the gunshot wound and mortality, p=0.017. We recommend the rigorous implementation of the Firearm Control Act and a national surveillance system for all fatal and non-fatal GSIs. There should be concerted efforts by Government and non-governmental organizations to create jobs and wealth thereby making crime less attractive.

## To the editors of the Pan African Medical Journal

The magnitude of civilian gunshot injuries has been compounded in sub-Saharan Africa due to civil wars in Libya, Somalia, Mali, Burkina Faso and Sudan [1]. These violent conflicts have contributed to the proliferation of firearms among insurgent groups, herders and armed robbers in Nigeria. Approval to conduct this study was obtained from the UI/UCH Ethical Committee (UI/EC/24/0698). We reviewed the medical records of all civilians with gunshot wounds at our hospital’s Emergency Department between 2014 and 2018 to determine the patient characteristics, pattern, and outcome. Sociodemographic variables and other parameters related to the firearm-related wound sustained, such as, age, gender, time of presentation, anatomical site of injury, gun characteristics, treatments offered, and treatment outcome were obtained. We defined outcome poor, if the patient was admitted for further management or demised, and good, if treated and discharged home. The relevant information was inputted by hand into a proforma designed for the study, coded, cleaned and entered into SPSS for windows (IBM Corp. Released 2013. IBM SPSS Statistics for Windows, Version 29.0. IBM Corporation, Armonk, NY, USA) and analyzed. A P-value of <0.05 was considered statistically significant.

The article by Afuwape *et al*. [2], in 2006 on civilian gunshot injuries at the University College Hospital, Ibadan and this 5-year audit buttress the fact that firearm-related trauma has remained a public health problem in Western Nigeria. Given that some cases would present at hospitals nearer to the injury sites and more severely wounded patients might have died enroute the ED, our high numbers (232) do not represent the overall casualties. Our numbers also topped the total cases (182) seen at a London major trauma center over 7 years [3]. However, reports from South Africa and the USA indicate a higher incidence than most international figures [4-6]. The number of GSI cases reported in 2016 doubled the numbers seen in 2014 and 2015. Given that there was a general election in Nigeria in 2015 with heightened security issues and proliferation of small firearms, the relationship between GSI and general elections in Nigeria should be explored further. However, in South Africa, Germany and the USA, homicides and suicides rank among the commonest causes of firearm injuries [5,7,8]. In our study, the upper and lower limbs were the most frequently injured anatomical sites, like the findings of researchers in developing and developed countries [4,6,9,10]. According to our data, there was an association between the injury site and mortality with gunshots to the head, chest, abdomen and multiple sites proving fatal. Although we recorded a low number of deaths (7.8%), we cannot make comparison with other studies because since we did not extend our observation beyond the ED, some deaths could have occurred among the 115 (50%) victims admitted to the hospital for further management. This is one notable limitation of our study. It has become imperative to set up an electronic record system which can easily link the ED database to the Ward records to obtain outcome data. Like every retrospective study, we do not lay claim to complete data, so our results may not be generalized.

**Table 1:** anatomical location of gunshot injury vis-à-vis patient mortality

Variable	Number with injury (n)	Mortality	Value*	p-value	Odds Ratio	95% CI
Head, Chest, Abdomen & Multiple injuries	118	14	5.656	0.017	3.702	1.18-11.61
**Upper limb, Lower limb & Back**	**114**	4			1	

* Chi square

**Figure 1:**
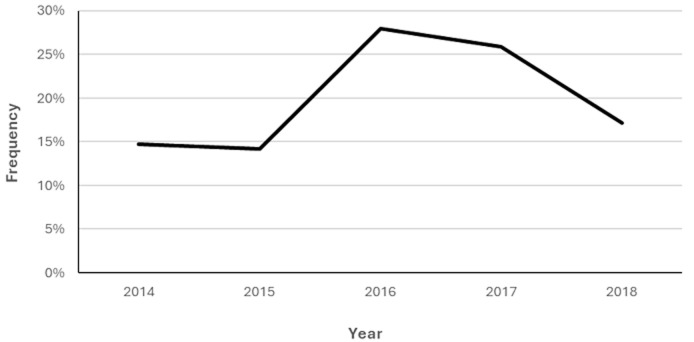
yearly trends of gunshot injuries during the study period

## Conclusion

Our study indicated that firearm-related injuries are prevalent, mostly due to armed robbery attacks, targeted at the young adult male population. There must be a rigorous implementation of the Firearm Control Act and a national surveillance system for all GSIs fatal and non-fatal. There should be concerted efforts by Government and non-governmental organizations to create jobs and wealth, which should ultimately make crime less attractive.
